# Effect of Mobile Phone Text Messaging Self-Management Support for Patients With Diabetes or Coronary Heart Disease in a Chronic Disease Management Program (SupportMe) on Blood Pressure: Pragmatic Randomized Controlled Trial

**DOI:** 10.2196/38275

**Published:** 2023-06-16

**Authors:** Ngai Wah Cheung, Julie Redfern, Aravinda Thiagalingam, Tien-Ming Hng, Simone Marschner, Rabbia Haider, Sonia Faruquie, Amy Von Huben, Shelley She, Daniel McIntyre, Jin-Gun Cho, Clara K Chow, The SupportMe Investigators

**Affiliations:** 1 Department of Diabetes & Endocrinology Westmead Hospital Westmead Australia; 2 Westmead Applied Research Centre Faculty of Medicine & Health University of Sydney Westmead Australia; 3 School of Health Sciences Faculty of Medicine & Health University of Sydney Sydney Australia; 4 Department of Cardiology Westmead Hospital Westmead Australia; 5 Blacktown Hospital Blacktown Australia; 6 Department of Respiratory Medicine Westmead Hospital Westmead Australia; 7 See Acknowledgments

**Keywords:** diabetes mellitus, type 2, coronary disease, chronic disease, SMS text messaging, delivery of health care, integrated, self-management

## Abstract

**Background:**

Maintaining engagement and support for patients with chronic diseases is challenging. SMS text messaging programs have complemented patient care in a variety of situations. However, such programs have not been widely translated into routine care.

**Objective:**

We aimed to examine the implementation and utility of a customized SMS text message–based support program for patients with type 2 diabetes (T2D), coronary heart disease, or both within a chronic disease integrated care program.

**Methods:**

We conducted a 6-month pragmatic parallel-group, single-blind randomized controlled trial that recruited people with T2D or coronary heart disease. Intervention participants received 4 semipersonalized SMS text messages per week providing self-management support to supplement standard care. Preprogrammed algorithms customized content based on participant characteristics, and the messages were sent at random times of the day and in random order by a fully automated SMS text messaging engine. Control participants received standard care and only administrative SMS text messages. The primary outcome was systolic blood pressure. Evaluations were conducted face to face whenever possible by researchers blinded to randomization. Participants with T2D were evaluated for glycated hemoglobin level. Participant-reported experience measures were evaluated using questionnaires and focus groups and summarized using proportions and thematic analysis.

**Results:**

A total of 902 participants were randomized (n=448, 49.7% to the intervention group and n=454, 50.3% to the control group). Primary outcome data were available for 89.5% (807/902) of the participants. At 6 months, there was no difference in systolic blood pressure between the intervention and control arms (adjusted mean difference=0.9 mm Hg, 95% CI −1.1 to 2.1; *P*=.38). Of 642 participants with T2D, there was no difference in glycated hemoglobin (adjusted mean difference=0.1%, 95% CI −0.1% to 0.3%; *P*=.35). Self-reported medication adherence was better in the intervention group (relative risk=0.82, 95% CI 0.68-1.00; *P*=.045). Participants reported that the SMS text messages were useful (298/344, 86.6%) and easily understood (336/344, 97.7%) and motivated change (217/344, 63.1%). The lack of bidirectional messaging was identified as a barrier.

**Conclusions:**

The intervention did not improve blood pressure in this cohort, possibly because of high clinician commitment to improved routine patient care as part of the chronic disease management program as well as favorable baseline metrics. There was high program engagement, acceptability, and perceived value. Feasibility as part of an integrated care program was demonstrated. SMS text messaging programs may supplement chronic disease management and support self-care.

**Trial Registration:**

Australian New Zealand Clinical Trials Registry ACTRN12616001689460; https://anzctr.org.au/Trial/Registration/TrialReview.aspx?id=371769&isReview=true

**International Registered Report Identifier (IRRID):**

RR2-10.1136/bmjopen-2018-025923

## Introduction

### Background

In 2015, a total of 71% of the 56 million global deaths were due to chronic diseases [1]. Coronary heart disease (CHD) and diabetes are 2 of the leading causes of chronic disease, together accounting for an estimated 41% of noncommunicable disease–related deaths of people aged <70 years [1]. Chronic diseases have long-lasting impacts on health and quality of life, as well as social and economic consequences [2,3]. It is common for people to have multiple chronic diseases.

SMS text messaging is a simple digital intervention that has been shown to improve behavioral risk factors for chronic disease. It is an accessible and affordable means of delivering health messages. Evidence for its use in smoking cessation has been well established through numerous randomized controlled trials (RCTs), and it is now a standard component of many smoking cessation programs [4,5]. Meta-analyses of clinical trials have shown the benefits of SMS text messaging for weight loss [6], physical activity [7], and medication adherence [8].

There is also evidence that SMS text messaging can be an effective adjunct to clinical care in the management of chronic diseases, including CHD and type 2 diabetes (T2D). The Tobacco, Exercise, and Diet Messages (TEXT ME) study demonstrated that an SMS text messaging program providing motivation, support, and education to people with CHD improved multiple clinical risk factor measures, including low-density lipoprotein cholesterol (LDL-C) level, blood pressure (BP), BMI, physical activity, and smoking cessation [9]. The program was cost-effective [10], engaging, useful, and easy to understand by patients [11]. Furthermore, in a post hoc analysis, the TEXT ME program was shown to specifically improve cardiovascular risk factors in the diabetes subgroup [12]. Among people with T2D, a meta-analysis of smaller SMS text messaging studies found that they achieved an overall reduction in glycated hemoglobin (HbA_1c_) level of 0.38% (4 mmol/mol) [13]. In total, 3 recent larger studies have similarly demonstrated modest improvements in HbA_1c_ level [14-16].

Apart from their use in smoking cessation, SMS text messaging programs have not been widely implemented or translated into routine health care for patients. Moreover, as outlined earlier in this section, previous trials have generally focused on a single disease or condition. It may be impractical or overwhelming for patients to be enrolled in separate programs for each of their chronic conditions. We do not know how well SMS text messaging programs can be integrated into routine clinical care and whether they can be structured to support people with multiple chronic conditions.

### Objectives

Therefore, we sought to understand whether a healthy lifestyle self-management support SMS text messaging program can complement a health district–wide integrated care program in terms of its effect on clinical measures as well as patient engagement. The primary goal of this implementation trial was to assess the effect of an SMS text messaging program for patients with 1 or 2 chronic health conditions, that is, T2D and CHD, in reducing BP. Secondary goals were to (1) develop an approach to integrating SMS text messaging program content suitable for patients with different or multiple chronic diseases; (2) examine the effect of the intervention on other clinical metrics; and (3) examine the feasibility, acceptability, barriers, and enablers of this program when implemented as part of a chronic disease integrated care program.

## Methods

### Trial Design

SupportMe was a 6-month pragmatic parallel design, single-blind RCT comparing participants with T2D or CHD supported by a simple digital health intervention program with participants receiving usual care.

The study was conducted in the Western Sydney Local Health District (WSLHD) in the Australian state of New South Wales. The WSLHD serves a population of a million inhabitants. In Western Sydney, 60% of the population is overweight or obese, and 5.9% is registered with diabetes compared with the national registered prevalence of 5.4% [17].

In 2014 to 2015, the WSLHD implemented the Western Sydney Integrated Care Program (WSICP) [18]. The WSICP was a government initiative developed in the WSLHD to improve the continuity of and access to care for people with the chronic diseases of CHD, T2D, and chronic obstructive pulmonary disease across hospital settings and primary care. It initially involved >50 clinicians in 2 teaching hospitals and 200 general practitioners (GPs) [18]. Components of the WSICP included standardization of care processes, the employment of nurse care coordinators to support patient care, rapid access to specialist support services for GPs, GP upskilling programs, and the use of IT for the sharing of data and care plans between hospital services and GPs [18]. The model of routinely embedding digital enablers to support people living with chronic diseases into the WSICP arose from stakeholder meetings involving health administrators, hospital clinicians (medical and allied health), GPs, public health physicians, and consumers.

### Participants

Recruitment for SupportMe was largely based on referrals from hospital specialist teams, community specialists and allied health staff, and GPs involved in the WSICP. Options for referral included a personal approach, fax, telephone, SMS text message, and email. Potential participants were given a detailed Participant Information and Consent Form to read, and every page of this needed to be acknowledged by being signed and witnessed. If enrolled, the referring clinician and GP were notified. Eligibility criteria were being aged ≥18 years, owning a mobile phone, being able to read SMS text messages in English, and having either CHD or suboptimally managed T2D. CHD was defined as previous myocardial infarction or documented >50% occlusion of a major coronary artery on coronary angiography, and suboptimally managed T2D was defined as having an HbA_1c_ level in the last 6 months of 7.1% to 11.4% (54-101 mmol/mol). Exclusion criteria were the inability to complete the study procedures or follow-up or having a condition that rendered the participant unsuitable for the study (eg, severe disability or a considerable memory or behavioral disorder). Participants with type 1 diabetes were not excluded but could only be included based on CHD, not diabetes, as the diabetes SMS text messages were designed for T2D, not type 1 diabetes. Participants needed to be able to read and understand SMS text messages in English. If needed, they were offered brief training at enrollment on how to read an SMS text message and delete or save messages.

### Interventions

The protocol for SupportMe has been previously described [19]. Both intervention and control participants were encouraged to receive usual care from their regular health professionals. Control participants received a welcome message and a reminder for their 6-month follow-up appointment only.

The intervention comprised 4 SMS text messages per week sent automatically at random times by an SMS text messaging engine between 9 AM and 5 PM on weekdays for 6 months. Messages were personalized (eg, including the participant’s name and the hospital they were connected to), customized based on baseline clinical characteristics and risk factors (eg, smoking, insulin use, and vegetarianism) and chronic disease type (CHD, T2D, or both), and selected as per prespecified computerized algorithms [10].

SMS text messages from a previous study [9] were adopted, and new content was developed through our iterative co-design process with investigators, specialist physicians, allied health professionals, and consumers [20,21]. The messages provided advice, motivation, information, or supportive tips on disease management. Each week, each of the four messages would address a different aspect: (1) general health, (2) nutrition, (3) physical activity, and (4) disease self-management. The distribution of messages is described in Table S1 in Multimedia Appendix 1. The messages within each category were sent to participants in random order.

Each message was unique. Examples of the SMS text messages include “Did you exercise today?” “Has your Dr checked & discussed your cholesterol levels with you recently? These need regular review,” “Healthy eating means at least 5 serves of vegetables & 2 serves of fruit every day,” “Did you know that some diabetes medications can help with weight loss? Talk to your Doctor to see if these are options for you,” “Activity can be accumulated in shorter bouts of 10 minutes each,” and “Try steaming, baking or grilling to reduce the need for oil when cooking.” The number of messages addressing various topics is provided in Table S1 in Multimedia Appendix 1. All messages concluded with a sign-off indicating that it was from the SupportMe program and the hospital team that the participant was primarily associated with.

Messaging was unidirectional with no expectation of return messages, but these were monitored by a researcher. Where return messages suggested potential clinical concern, they were escalated to a physician for review. This researcher did not participate in the data collection or analysis.

### Randomization

Computerized randomization was conducted in-house using a randomization function from the Xojo framework (Xojo, Inc). This occurred with a uniform allocation of 1:1 (block size 8) stratified by health condition (T2D, CHD, or both). Randomization automatically occurred following the baseline study visit and entry of baseline data into a secure REDCap (Research Electronic Data Capture; Vanderbilt University) web interface. The computerized platform connected to the SMS text messaging platform to send messages to the intervention participants automatically based on randomization. To minimize unblinding at follow-up, all participants were sent a message requesting them not to reveal treatment allocation to the data collectors.

### Blinding

The study was single-blinded. Participants were aware of whether they had received the SMS text messaging intervention. However, the study personnel collecting the data and the statistician were blinded to group allocation.

### Data Collection

Participants were assessed face to face at baseline and at 6 months. This occurred in hospital and private clinic settings. BP, heart rate, weight, height, and waist circumference were measured. Questionnaires assessed physical activity (Global Physical Activity Questionnaire) [22], dietary intake (combination of the dietary component of the World Health Organization stepwise approach to noncommunicable disease risk factor surveillance and the diet questionnaire from the TEXT ME study [Multimedia Appendix 2]) [9,23], quality of life (12-item Short Form Health Survey) [24], depression (Patient Health Questionnaire–9 depression scale) [25], smoking status, and medication use.

The Global Physical Activity Questionnaire collects physical activity data in 3 domains: activity at work, travel to and from places, and recreational activity. The work and recreational activity domains ask about vigorous- and moderate-intensity activities, whereas the travel domain only records moderate-intensity activity. The total physical activity is given as the number of metabolic equivalent of task (MET) minutes per week. This was calculated by multiplying the total minutes of vigorous activity undertaken in a typical week by 8 and the total minutes of moderate-intensity activity undertaken per week by 4 and then adding these 2 figures [22].

Our dietary questionnaire was a food frequency questionnaire that assessed the consumption of fruits, vegetables, fish, oil, and salt (Multimedia Appendix 2). Weekly fruit consumption was determined by multiplying the number of servings eaten on a typical day by the number of days in a typical week in which fruit was eaten. Weekly vegetable consumption was determined by multiplying the number of servings eaten on a typical day by the number of days in a typical week in which vegetables were eaten. Fish consumption was determined by asking how many grams of fish were eaten in a typical week. The questionnaire also asked how many meals per week were eaten that were not prepared at home.

The SF-12 was used to assess quality of life, with physical and mental components. A score was generated by applying the proprietary algorithm used by QualityMetric [14]. Higher scores indicate better physical and mental health functioning. The Patient Health Questionnaire–9 depression scale was used to assess the depression score. It comprises 9 Likert-scale questions ranging from 0 to 3 that are summed so that the minimum score is 0 and the maximum score is 27. A higher score indicates a greater degree of depression, with a score of 1 to 4 indicating minimal depression and a score of 20 to 27 being indicative of severe depression.

Fasting total cholesterol, LDL-C, high-density lipoprotein cholesterol, triglyceride, and fasting glucose levels were obtained, as well as HbA_1c_ level for those with diabetes. For participants unable to return for the 6-month assessment, partial data collection was obtained via telephone and review of medical records. As this was a pragmatic trial, routine blood tests organized by participants’ health professionals were accepted.

Serious adverse events related to diabetes or CHD were adjudicated by physicians independently from the study.

### Outcomes

Systolic BP (SBP) was chosen as the primary outcome at 6 months as this is an important modifiable risk factor common to patients with both CHD and T2D. Secondary outcomes included diastolic BP (DBP), BMI, waist circumference, fasting LDL-C level, fasting glucose level, physical activity, dietary intake, quality of life, depression score, smoking cessation, and if any medications were missed in the previous 30 days. Participants with T2D were evaluated for HbA_1c_ level.

### Process Evaluation

We followed our previous methodology of process evaluation [11]. The implementation of the intervention was monitored using screening logs along with a log of study interactions with participants. At the 6-month time point, all intervention participants were administered a survey that explored the acceptability, suitability, engagement, and perceived utility of the SMS text messages.

Participants allocated to the intervention group (who provided consent to participate in further research) were also consecutively invited to participate in a focus group discussion. The number of focus groups was determined by thematic saturation, with a target of 6 to 10 participants per focus group. The in-person focus group discussions (1.5-hour duration) were conducted according to usual processes, were facilitated by an experienced researcher (RH), and were audio recorded and transcribed. Focus groups were facilitated according to a discussion guide that explored key topics, including participant perceptions of the texting program and its utility along with barriers to and enablers of its implementation (Table S2 in Multimedia Appendix 1). For the analysis, the demographic information of the focus group participants was summarized using means and proportions. The analysis was thematic, with transcriptions coded by a clinician researcher. Representative direct quotes were then selected to illustrate themes.

The study planned to recruit 1000 participants as sample size calculations estimated that this number would enable detection of a 3.5 mm Hg difference in SBP with 80% power and 20% loss to follow-up (type-1 error of 5% and 2-sided α assuming an SD of 17 mm Hg). A sample size of 625 patients with diabetes allowing for 20% dropout had 80% power to detect a difference of 0.3% in HbA_1c_ level (SD of 1.2% based on local data [26]).

### Statistical Methods

We followed a prespecified statistical analysis plan and intention-to-treat principles. Baseline continuous variables were presented as means and SDs. Survey data were summarized by proportions. Outcome comparisons were presented as adjusted means or relative risks, and 95% CIs between treatment groups were calculated using regression models adjusting for the baseline measure of that specific outcome. For dichotomous outcomes, log-binomial regression was used, and for continuous outcomes, linear regression was used. Analyses were conducted using R (version 3.5.2; R Foundation for Statistical Computing) packages. Statistical tests were 2-tailed, with a 5% significance threshold.

Although endeavoring to capture outcome data within 1 month of the 6-month time point, we prespecified that results obtained 4 to 13 months after randomization were acceptable but as protocol deviations. As this was a pragmatic trial, the exact timing of the outcome assessment could not be controlled as it was based on routine care. A sensitivity analysis that restricted the data to results from 5 to 8 months after randomization found no impact on the final conclusions.

Differences in the effect of the intervention on the primary outcome (SBP) for subgroups were explored using prespecified risk factors. A similar prespecified analysis was undertaken for HbA_1c_ level. Subgroup analyses were conducted for the participants with diabetes and for those with CHD.

### Ethics Approval

This study was registered with the Australian New Zealand Clinical Trials Registry (ACTRN12616001689460) and approved by the WSLHD Human Research Ethics Committee (AU RED HREC/16/WMEAD/331). All participants provided written consent.

## Results

### Participant Characteristics

**Figure 1:**
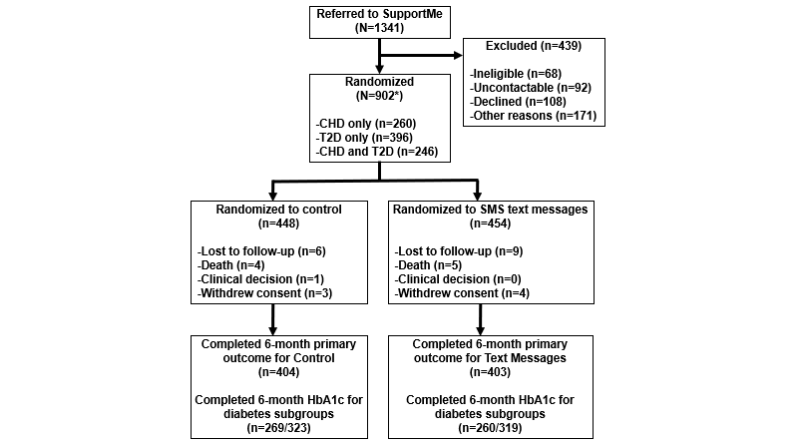
CONSORT (Consolidated Standards of Reporting Trials) diagram for the SupportMe randomized controlled trial. *Excluding 1 participant who was inadvertently randomized twice. CHD: coronary heart disease; HbA_1c_: glycated hemoglobin; T2D: type 2 diabetes.

Between May 2017 and April 2019, a total of 1341 participants were referred to the study, of whom 439 (32.74%) were excluded as they did not meet all study criteria or declined to participate and 902 (67.26%) were randomized (n=448, 49.7% to the intervention group and n=454, 50.3% to the control group). A total of 96.5% (870/902) of participants reached the 6-month follow-up with at least partial data completion, and primary outcome data were obtained for 89.5% (807/902) of the participants (Figure 1). Recruitment ceased early because of budget constraints and meeting the number of participants with diabetes as per the secondary sample size calculations.

Of the 902 participants, 260 (28.8%) received the CHD-only program, 396 (43.9%) received the T2D-only program, and 246 (27.3%) received the CHD and T2D program. A total of 16.5% (43/260) of participants with T2D did not meet the HbA_1c_ range for study inclusion but had CHD, so they received the CHD-only program. There were 0.4% (4/902) of participants who had type 1 diabetes. They were included in the CHD-only program. The mean participant age was 61.5 (SD 11.6) years, and 28.5% (257/902) were women. The mean baseline SBP was 129 (SD 17) mm Hg, mean baseline DBP was 78 (SD 10) mm Hg, mean baseline BMI was 32.0 (SD 7.1) kg/m^2^, mean baseline LDL-C level was 2.0 (SD 0.9) mmol/L, mean baseline fasting glucose level was 8.2 (SD 3.2) mmol/L, and mean baseline HbA_1c_ level was 8.4% (SD 1.3%). Baseline characteristics were similar between the groups (Table 1).

**Table 1:** Baseline characteristics by treatment group (n=902).

		Control (n=448)	Intervention (n=454)	Total
Age (years), mean (SD)		61.9 (12.0)	61.0 (11.1)	61.5 (11.6)
**Sex, n (%)**				
	Female	128 (28.6)	129 (28.4)	257 (28.5)
	Male	320 (71.4)	325 (71.6)	645 (71.5)
**Ethnicity, n (%)**				
	Australian or New Zealander	213 (47.5)	233 (51.3)	446 (49.4)
	Asian	106 (23.7)	88 (19.4)	194 (21.5)
	Other	129 (28.8)	133 (29.3)	262 (29)
**Marital status, n (%)**				
	Married or de facto partnership	329 (73.4)	315 (69.4)	644 (71.4)
	Single, separated, or widowed	119 (26.6)	139 (30.6)	258 (28.6)
Years of formal education, mean (SD)		13.5 (3.8)	13.7 (4.1)	13.6 (4.0)
**Employment status, n (%)**				
	Working full time	148 (33)	152 (33.5)	300 (33.3)
	Working part-time	45 (10)	43 (9.5)	88 (9.8)
	Not working	255 (56.9)	259 (57)	514 (57)
**History of diabetes, n (%)**				
	Type 1 diabetes	3 (0.7)	1 (0.2)	4 (0.4)
	Type 2 diabetes	341 (76.1)	344 (75.8)	685 (75.9)
**History of CHD^a^, n (%)**				
	All CHD	258 (57.6)	262 (57.7)	520 (57.6)
	Myocardial infarction	149 (57.8)^b^	160 (61.1)^c^	309 (59.4)^d^
	CABGs^e^	74 (28.7)^b^	68 (26)^c^	142 (27.3)^d^
	PCI^f^	154 (59.7)^b^	163 (62.2)^c^	317 (61)^d^
Family history of CHD, n (%)		241 (54)^g^	263 (58.4)^h^	504 (56.3)^i^
Family history of diabetes, n (%)		256 (57.1)	274 (61)^j^	530 (59.1)^k^
**Referral source, n (%)**				
	Specialist or hospital	364 (81.4)^l^	369 (81.3)	733 (81.4)^m^
	GP^n^ or community	83 (18.6)^l^	85 (18.7)	168 (18.6)^m^
Current smoker, n (%)		39 (8.8)^o^	35 (7.8)^h^	74 (8.3)^p^
Current drinker, n (%)		226 (50.8)^o^	256 (56.9)^h^	482 (53.9)^p^
Exercises regularly, n (%)		169 (37.7)	183 (40.3)	352 (39)
Total physical activity (MET^q^ minutes per week), mean (SD)		1895 (3177)	1902 (3591)	1898 (3388)
SBP^r^ (mm Hg), mean (SD)		129.8 (17.6)	128.0 (16.7)	128.9 (17.2)
DBP^s^ (mm Hg), mean (SD)		78.5 (10.8)	77.6 (10.0)	78.0 (10.4)
BMI (kg/m^2^), mean (SD)		31.6 (7.1)	32.4 (7.2)	32.0 (7.1)
Waist circumference (cm), mean (SD)		109.1 (16.2)	111.4 (17.1)	110.3 (16.7)
Heart rate, mean (SD)		74.5 (12.8)	74.0 (13.0)	74.3 (12.9)
LDL^t^ cholesterol (mmol/L), mean (SD)		2.0 (0.9)	2.0 (0.9)	2.0 (0.9)
HDL^u^ cholesterol (mmol/L), mean (SD)		1.1 (0.3)	1.1 (0.3)	1.1 (0.3)
Total cholesterol (mmol/L), mean (SD)		4.0 (1.2)	4.0 (1.2)	4.0 (1.2)
Triglycerides (mmol/L), mean (SD)		2.1 (2.1)	2.0 (1.4)	2.0 (1.8)
Fasting glucose (mmol/L), mean (SD)		8.4 (3.5)	8.0 (3.0)	8.2 (3.2)
HbA_1c_^v^ (%), mean (SD)		8.4 (1.3)	8.4 (1.3)	8.4 (1.3)
HbA_1c_^v^ (mmol/mol), mean (SD)		68 (14)	68 (14)	68 (14)
**Antihypertensive and diabetes medication, n (%)**				
	ACE^w^ inhibitors	130 (29)	147 (32.4)	277 (30.7)
	Angiotensin 2 receptor blockers	132 (29.5)	128 (28.2)	260 (28.8)
	Thiazide diuretics	39 (8.7)	43 (9.5)	82 (9.1)
	β-blockers	167 (37.3)	178 (39.2)	345 (38.2)
	Calcium channel blockers	95 (21.2)	106 (23.3)	201 (22.3)
	Other antihypertensives	61 (13.6)	81 (17.8)	142 (15.7)
	Metformin	262 (58.5)	275 (60.6)	537 (59.5)
	Sulfonylureas	85 (19)	81 (17.8)	166 (18.4)
	DPP^x^-4 inhibitors	66 (14.7)	56 (12.3)	122 (13.5)
	SGLT^y^-2 inhibitors	76 (17)	101 (22.2)	177 (19.6)
	α-glucosidase	1 (0.2)	4 (0.9)	5 (0.6)
	GLP^z^-1 agonists	39 (8.7)	31 (6.8)	70 (7.8)
	Insulin	166 (37.1)	163 (35.9)	329 (36.5)

^a^CHD: coronary heart disease.

^b^n=258.

^c^n=262.

^d^n=520.

^e^CABG: coronary artery bypass graft.

^f^PCI: percutaneous coronary intervention.

^g^n=446.

^h^n=450.

^i^n=896.

^j^n=449.

^k^n=897.

^l^n=447.

^m^n=901.

^n^GP: general practice.

^o^n=445.

^p^n=895.

^q^MET: metabolic equivalent of task.

^r^SBP: systolic blood pressure.

^s^DBP: diastolic blood pressure.

^t^LDL: low-density lipoprotein.

^u^HDL: high-density lipoprotein.

^v^HbA_1c_: glycated hemoglobin; performed for participants with diabetes only.

^w^ACE: angiotensin-converting enzyme.

^x^DPP: dipeptidyl peptidase.

^y^SGLT: sodium-glucose cotransporter.

^z^GLP: glucagon-like peptide.

### Effect of the Intervention on the Primary Outcome

**Figure 2:**
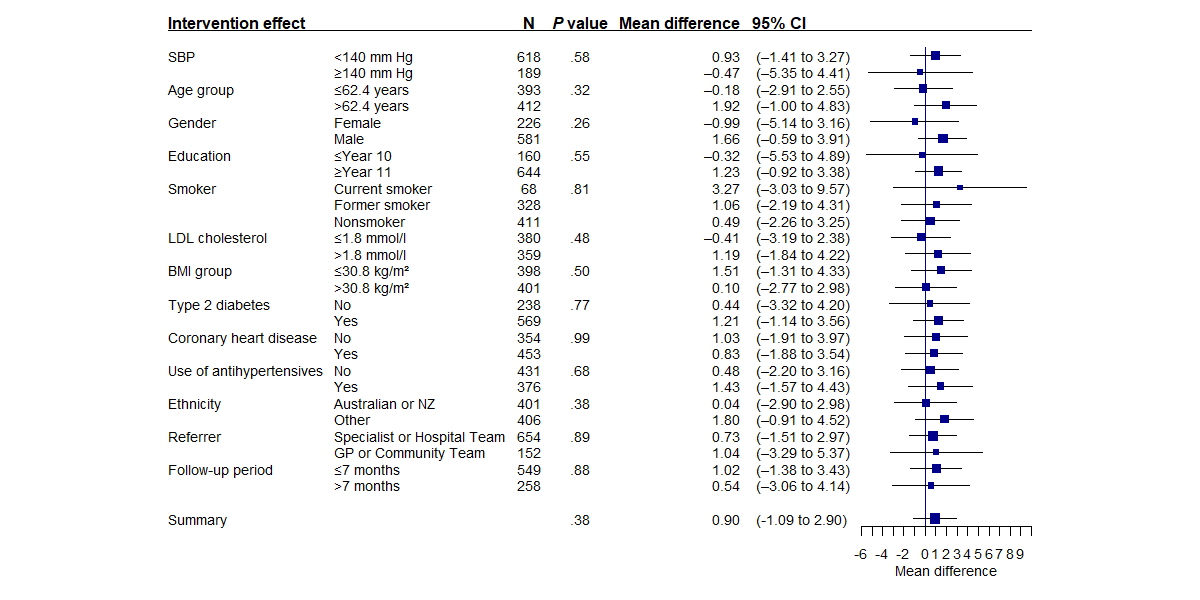
Interaction between subgroups by treatment group for the primary outcome (systolic blood pressure [SBP]). GP: general practitioner; LDL: low-density lipoprotein; NZ: New Zealander.

There was no difference in SBP between the groups at the 6-month assessment when adjusted for baseline SBP (adjusted mean difference=0.9 mm Hg, 95% CI −1.1 to 2.9; *P*=.38; Table 2). The prespecified analyses demonstrated no treatment interaction with baseline SBP, diabetes status, CHD status, LDL-C level, age, gender, education status, smoking status, BMI, or ethnicity (Figure 2).

**Table 2:** Primary and secondary outcomes at the 6-month assessment. Analyses for each outcome were adjusted for the baseline measure of that specific outcome (n=807).

		Control (n=404)	Intervention (n=403)	Adjusted mean difference^a^ or relative risk^b^ (95% CI)	*P* value
**Primary outcome**					
	Systolic blood pressure (mm Hg), adjusted mean (95% CI)	127.9 (126.5 to 129.3)	128.8 (127.4 to 130.2)	0.90 (−1.1 to 2.9)^a^	.38
**Secondary outcomes**					
	BMI (kg/m^2^), adjusted mean (95% CI)	31.8 (31.6 to 31.9)	31.6 (31.5 to 31.8)	−0.1 (−0.4 to 0.1)^a^	.25
	Diastolic blood pressure (mm Hg), adjusted mean (95% CI)	77.0 (76.1 to 77.9)	77.4 (76.5 to 78.3)	−0.2 (−1.6 to 1.3)^a^	.62
	Waist circumference (cm), adjusted mean (95% CI)	109.5 (108.8 to 110.2)	109.2 (108.5 to 109.9)	−0.2 (−1.2 to 0.8)^a^	.67
	LDL^c^ cholesterol (mmol/L), adjusted mean (95% CI)	1.9 (1.8 to 2.0)	1.9 (1.9 to 2.0)	0.0 (−0.1 to 0.2)^a^	.56
	Fasting glucose (mmol/L), adjusted mean (95% CI)	7.6 (7.3 to 7.9)	7.7 (7.4 to 8.1)	0.2 (−0.3 to 0.6)^a^	.55
	HbA_1c_^d^ (%)^e^, adjusted mean (95% CI)	7.7 (7.6 to 7.9)	7.8 (7.7 to 8.0)	0.1 (−0.1 to 0.3)^a^	.35
	HbA_1c_ (mmol/mol)^e^, adjusted mean (95% CI)	61 (60 to 63)	62 (61 to 64)	1 (−1 to 3)^a^	.35
	Total physical activity (MET^f^ minutes per week), adjusted mean (95% CI)	1793 (1524 to 2060)	2035 (1766 to 2304)	242 (−137 to 621)^a^	.21
	Fruit consumption (servings per week), adjusted mean (95% CI)	10.8 (10.1 to 11.5)	11.4 (10.8 to 12.1)	0.6 (−0.3 to 1.6)^a^	.20
	Vegetable consumption (servings per week), adjusted mean (95% CI)	15.2 (14.2 to 16.3)	16.1 (15.1 to 17.2)	0.9 (−0.6 to 2.3)^a^	.23
	Fish consumption (grams per week), adjusted mean (95% CI)	194.6 (174.5 to 214.7)	235.9 (215.8 to 256.1)	41.3 (12.9 to 69.8)^a^	.004
	Meals not prepared at home eaten per week, adjusted mean (95% CI)	1.7 (1.5 to 1.9)	1.7 (1.5 to 1.9)	−0.0 (−0.3 to 0.3)^a^	.79
	Using butter or ghee for cooking, n (%)	294 (78.8)^g^	270 (73)^h^	0.9 (0.9 to 1.0)^b^	.05
	Continued current smokers^i^, n (%)	26 (78.8)^j^	21 (70)^k^	0.9 (0.7 to 1.2)^b^	.42
	Quality of life: physical component summary, adjusted mean (95% CI)	46.5 (45.7 to 47.2)	46.6 (45.8 to 47.3)	0.1 (−0.9 to 1.2)^a^	.82
	Quality of life: mental component summary, adjusted mean (95% CI)	48.8 (48.3 to 49.4)	49.3 (48.8 to 49.9)	0.5 (−0.3 to 1.3)^a^	.24
	Depression score (PHQ-9^l^), adjusted mean (95% CI)	5.6 (5.2 to 6.1)	5.5 (5.0 to 5.9)	−0.1 (−0.8 to 0.5)^a^	.67
	Missed medication on at least one of the last 30 days, n (%)	134 (36.1)^m^	110 (29.7)^h^	0.8 (0.7 to 1.0)^b^	.045

^a^Adjusted mean difference.

^b^Relative risk.

^c^LDL: low-density lipoprotein.

^d^HbA_1c_: glycated hemoglobin.

^e^Of 642 participants with type 2 diabetes and baseline HbA_1c_ of 7.1% to 11.4%.

^f^MET: metabolic equivalent of task.

^g^n=373.

^h^n=370.

^i^Of 63 participants who were current smokers at baseline.

^j^n=33.

^k^n=30.

^l^PHQ-9: Patient Health Questionnaire–9.

^m^n=371.

### Effect of the Intervention on the Secondary Outcomes

There were no significant differences between the groups in BMI, waist circumference, DBP, LDL-C level, and fasting glucose level at the 6-month assessment (Table 2). There was also no difference between the groups in physical activity, diet, depressive symptoms, and quality of life. Fewer participants in the intervention group reported missed medication on at least one of the last 30 days (relative risk=0.82, 95% CI 0.68-1.00; *P*=.045). There was greater fish consumption in the intervention group by 41 g per week (95% CI 12-70; *P*=.004).

### T2D Subgroup Analysis

**Figure 3:**
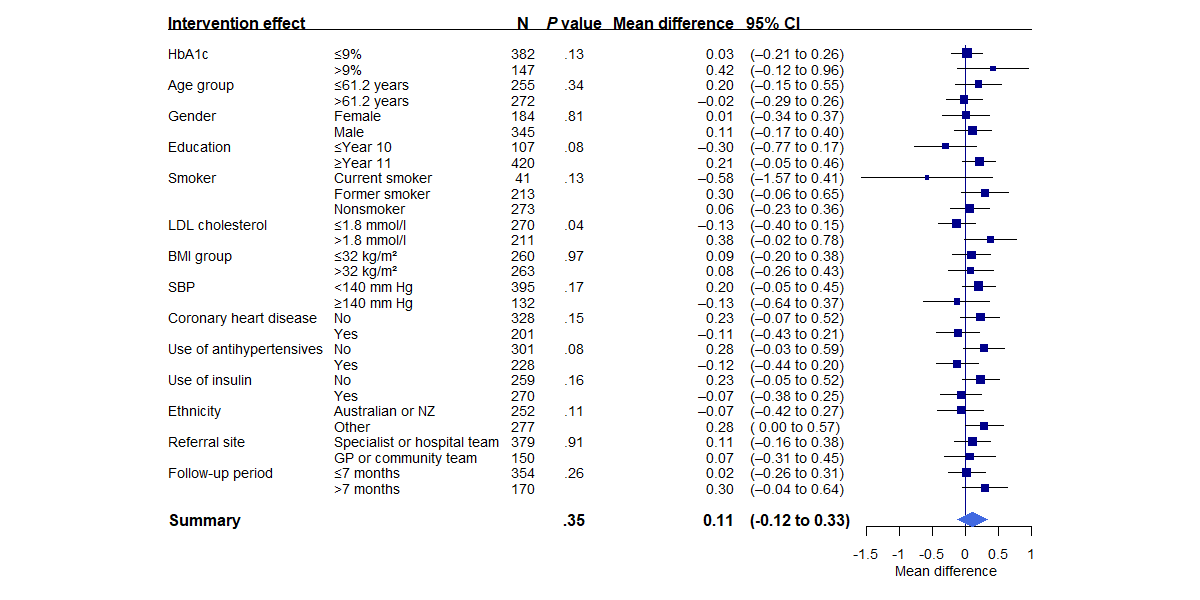
Interaction between subgroups by treatment group for the glycated hemoglobin (HbA_1c_) results at the 6-month assessment among participants with suboptimally controlled type 2 diabetes. GP: general practitioner; LDL: low-density lipoprotein; NZ: New Zealander; SBP: systolic blood pressure.

A subgroup analysis was conducted to examine the effect of the intervention on people with T2D, that is, those in the T2D and combined T2D and CHD groups. The characteristics of the participants with T2D are listed in Table 3, and the outcomes of the trial in this group are detailed in Table 4. There was no difference in the primary outcome (SBP) between the intervention and control groups at the 6-month assessment (adjusted mean difference=1.2 mm Hg, 95% CI −1.1 to 3.6; *P*=.31). There was a reduction in mean HbA_1c_ level from 8.4% (SD 1.3%) at baseline to 7.7% (SD 1.4%) in the intervention participants and to 7.8% (SD 1.4%) in the control participants (reduction from mean 68, SD 14 mmol/mol to mean 61, SD 15 mmol/mol and mean 62, SD 15 mmol/mol, respectively; Table 3). However, the difference in HbA_1c_ level at the 6-month assessment between the intervention and control arms was not significant (adjusted mean difference=0.1%, 95% CI −0.1% to 0.3%; *P*=.35). Prespecified analyses related to HbA_1c_ level did not demonstrate that the intervention was more effective for any subgroup based on baseline HbA_1c_ level, CHD, SBP, LDL-C level, age, gender, education status, smoking status, BMI, or ethnicity (Figure 3).

The increase in fish intake remained significant in the T2D subgroup analysis. There was no difference in missed medication between the intervention and control participants.

**Table 3:** Baseline characteristics of participants with type 2 diabetes by treatment group (n=642).

		Control (n=323)	Intervention (n=319)	Total
Age (years), mean (SD)		60.7 (11.8)	59.7 (11.0)	60.2 (11.4)
**Sex, n (%)**				
	Female	110 (34.1)	115 (36.1)	225 (35)
	Male	213 (65.9)	204 (63.9)	417 (65)
**Ethnicity, n (%)**				
	Australian or New Zealander	141 (43.7)	158 (49.5)	299 (46.6)
	Asian	87 (26.9)	65 (20.4)	152 (23.7)
	Other	95 (29.4)	96 (30.1)	191 (29.8)
**Marital status, n (%)**				
	Married or de facto partnership	229 (70.9)	213 (66.8)	442 (68.8)
	Single, separated, or widowed	94 (29.1)	106 (33.2)	200 (31.2)
Years of formal education, mean (SD)		13.5 (3.7)	13.4 (3.9)	13.5 (3.8)
**Employment status, n (%)**				
	Working full time	105 (32.5)	105 (32.9)	210 (32.7)
	Working part-time	38 (11.8)	28 (8.8)	66 (10.3)
	Not working	180 (55.7)	186 (58.3)	366 (57)
**History of CHD^a^, n (%)**				
	All CHD	133 (41.2)	127 (39.8)	260 (40.5)
	Myocardial infarction	72 (54.1)^b^	82 (64.6)^c^	154 (59.2)^d^
	CABGs^e^	40 (30.1)^b^	38 (29.9)^c^	78 (30)^d^
	PCI^f^	72 (54.1)^b^	76 (59.8)^c^	148 (56.9)^d^
Family history of CHD, n (%)		164 (50.9)^g^	175 (55.4)^h^	339 (53.1)^i^
Family history of diabetes, n (%)		218 (67.5)	227 (71.8)^h^	445 (69.6)^j^
Had an eye check in the last 6 months?, n (%)		207 (64.5)^k^	203 (64)^l^	410 (64.3)^i^
Number of times per week they monitor glucose levels, mean (SD)		12.3 (11.6)	14.9 (13.4)	13.6 (12.6)
**Referral source, n (%)**				
	Specialist or hospital	281 (87)	277 (86.8)	558 (86.9)
	GP^m^ or community	42 (13)	42 (13.2)	84 (13.1)
Current smoker, n (%)		26 (8.1)^g^	25 (7.9)^n^	51 (8)^o^
Current drinker, n (%)		155 (48.1)^g^	166 (52.2)^n^	321 (50.2)^o^
Exercises regularly, n (%)		115 (35.7)^g^	113 (35.4)^n^	228 (35.6)^o^
Total physical activity (MET^p^ minutes per week), mean (SD)		1895 (3177)	1902 (3591)	1898 (3388)
SBP^q^ (mm Hg), mean (SD)		130.3 (18.3)	128.3 (15.9)	129.3 (17.2)
DBP^r^ (mm Hg), mean (SD)		78.9 (10.6)	77.7 (9.7)	78.3 (10.2)
BMI (kg/m^2^), mean (SD)		32.7 (7.4)	33.4 (7.6)	33.1 (7.5)
Waist circumference (cm), mean (SD)		111.1 (16.3)	114.0 (17.6)	112.5 (17.0)
Heart rate, mean (SD)		76.8 (12.3)	76.1 (12.7)	76.5 (12.5)
LDL^s^ cholesterol (mmol/L), mean (SD)		2.0 (0.9)	1.9 (0.9)	2.0 (0.9)
HDL^t^ cholesterol (mmol/L), mean (SD)		1.1 (0.3)	1.1 (0.3)	1.1 (0.3)
Total cholesterol (mmol/L), mean (SD)		4.1 (1.2)	4.0 (1.2)	4.1 (1.2)
Triglycerides (mmol/L), mean (SD)		2.2 (2.4)	2.1 (1.5)	2.2 (2.0)
Fasting glucose (mmol/L), mean (SD)		9.3 (3.5)	9.1 (3.0)	9.2 (3.3)
HbA_1c_^u^ (%), mean (SD)		8.5 (1.3)	8.5 (1.4)	8.5 (1.3)
HbA_1c_ (mmol/mol), mean (SD)		68 (14)	68 (14)	68 (14)
**Antihypertensive and diabetes medication, n (%)**				
	ACE^v^ inhibitors	82 (25.4)	88 (27.6)	170 (26.5)
	Angiotensin 2 receptor blockers	104 (32.2)	94 (29.5)	198 (30.8)
	Thiazide diuretics	32 (9.9)	35 (11)	67 (10.4)
	β-blockers	101 (31.3)	106 (33.2)	207 (32.2)
	Calcium channel blockers	74 (22.9)	70 (21.9)	144 (22.4)
	Other antihypertensives	43 (13.3)	61 (19.1)	104 (16.2)
	Metformin	247 (76.5)	256 (80.3)	503 (78.3)
	Sulfonylureas	81 (25.1)	80 (25.1)	161 (25.1)
	DPP^w^-4 inhibitors	62 (19.2)	54 (16.9)	116 (18.1)
	SGLT^x^-2 inhibitors	75 (23.2)	99 (31)	174 (27.1)
	α-glucosidase	1 (0.3)	4 (1.3)	5 (0.8)
	GLP^y^-1 agonists	38 (11.8)	29 (9.1)	67 (10.4)
	Insulin	158 (48.9)	159 (49.8)	317 (49.4)

^a^CHD: coronary heart disease.

^b^n=133.

^c^n=127.

^d^n=260.

^e^CABG: coronary artery bypass graft.

^f^PCI: percutaneous coronary intervention.

^g^n=322.

^h^n=316.

^i^n=638.

^j^n=639.

^k^n=321.

^l^n=317.

^m^GP: general practice.

^n^n=318.

^o^n=640.

^p^MET: metabolic equivalent of task.

^q^SBP: systolic blood pressure.

^r^DBP: diastolic blood pressure.

^s^LDL: low-density lipoprotein.

^t^HDL: high-density lipoprotein.

^u^HbA_1c_: glycated hemoglobin.

^v^ACE: : angiotensin-converting enzyme.

^w^DPP: dipeptidyl peptidase.

^x^SGLT: sodium-glucose cotransporter.

^y^GLP: glucagon-like peptide.

**Table 4:** Primary and secondary outcomes at the 6-month assessment for type 2 diabetes (n=569).

			Control (n=292)^a^		Intervention (n=277)^a^		Adjusted mean difference^b^ or relative risk^c^ (95% CI)		*P* value
**Primary outcome**									
	Systolic blood pressure (mm Hg), adjusted mean (95% CI)	128.5 (126.8 to 130.1)		129.7 (128.0 to 131.4)		1.2 (−1.1 to 3.6)^b^		.31	
**Secondary outcomes**									
	HbA_1c_^d^, adjusted mean (95% CI)	7.7 (7.6 to 7.9)		7.8 (7.7 to 8.0)		0.1 (−0.1 to 0.3)		.35	
	BMI (kg/m^2^), adjusted mean (95% CI)	32.9 (32.7 to 33.1)		32.6 (32.5 to 32.8)		−0.2 (−0.5 to 0.1)^b^		.11	
	Diastolic blood pressure (mm Hg), adjusted mean (95% CI)	77.0 (75.9 to 78.1)		77.6 (76.5 to 78.7)		0.6 (−0.9 to 2.1)^b^		.47	
	Waist circumference (cm), adjusted mean (95% CI)	111.7 (110.8 to 112.6)		111.2 (110.3 to 112.1)		−0.5 (−1.7 to 0.7)^b^		.40	
	LDL^e^ cholesterol (mmol/L), adjusted mean (95% CI)	1.9 (1.8 to 2.0)		2.0 (1.9 to 2.1)		0.0 (−0.1 to 0.2)^b^		.73	
	Fasting glucose (mmol/L), adjusted mean (95% CI)	8.4 (8.0 to 8.9)		8.6 (8.2 to 9.1)		0.2 (−0.4 to 0.8)^b^		.55	
	Total physical activity (MET^f^ minutes per week), adjusted mean (95% CI)	1645 (1313 to 1966)		2039 (1706 to 2370)		393 (−68 to 854)^b^		.10	
	Fruit consumption (servings per week), adjusted mean (95% CI)	11.0 (10.1 to 11.8)		11.5 (10.7 to 12.4)		0.6 (−0.6 to 1.7)^b^		.35	
	Vegetable consumption (servings per week), adjusted mean (95% CI)	15.1 (13.9 to 16.2)		15.5 (14.4 to 16.7)		0.4 (−1.1 to 2.0)^b^		.58	
	Fish consumption (grams per week), adjusted mean (95% CI)	195.7 (172.1 to 218.5)		231.3 (207.7 to 254.9)		35.6 (2.8 to 68.3)^b^		.03	
	Meals not prepared at home eaten per week, adjusted mean (95% CI)	1.7 (1.5 to 1.9)		1.6 (1.4 to 1.9)		0.1 (−0.4 to 0.3)^b^		.65	
	Using butter or ghee for cooking, n (%)	214 (78.4)^g^		189 (74.1)^h^		0.9 (0.9 to 1.0)^c^		.09	
	Had eye check in the last 6 months, n (%)	250 (82.2)^i^		250 (85.3)^j^		1.1 (0.9 to 1.4)^c^		.31	
	Continued current smokers, n (%)	18 (81.8)^k^		18 (85.7)^l^		1.1 (0.81 to 1.4)^c^		>.99	
	Quality of life: physical component summary, adjusted mean (95% CI)	45.6 (44.8 to 46.5)		45.8 (45.0 to 46.7)		0.2 (−1.0 to 1.4)^b^		.77	
	Quality of life: mental component summary, adjusted mean (95% CI)	48.7 (48.0 to 49.4)		49.3 (48.6 to 50.0)		0.6 (−0.4 to 1.6)^b^		.23	
	Depression score (PHQ-9^m^), adjusted mean (95% CI)	6.1 (5.5 to 6.6)		5.9 (5.3 to 6.5)		−0.2 (−1.0 to 0.6)^b^		.64	
	Missed medication on at least one of the last 30 days, n (%)	101 (34.6)		83 (30)		0.9 (0.7 to 1.1)^c^		.30	

^a^Number of participants with data for primary outcome.

^b^Adjusted mean difference.

^c^Relative risk.

^d^HbA_1c_: glycated hemoglobin.

^e^LDL: low-density lipoprotein.

^f^MET: metabolic equivalent of task.

^g^n=273.

^h^n=255.

^i^n=304.

^j^n=293.

^k^n=22.

^l^n=21.

^m^PHQ-9: Patient Health Questionnaire–9.

### CHD Subgroup Analysis

A subgroup analysis was conducted to examine the effect of the intervention on people with CHD, that is, those in the CHD and combined T2D and CHD groups. The characteristics of the participants with CHD are listed in Table 5, and the outcomes of the trial in this group are detailed in Table 6. There was no difference in the primary outcome (SBP) between the intervention and control groups at the 6-month assessment (adjusted mean difference=0.8 mm Hg, 95% CI −1.9 to 3.5; *P*=.55).

The increase in fish intake remained significant in the CHD subgroup analysis. There was also a decrease in the use of butter or ghee for cooking. The reduction in missed medication was borderline significant (*P*=.05).

**Table 5:** Baseline characteristics of participants with coronary heart disease (CHD) by treatment group (n=506).

			Control (n=250)		Intervention (n=256)		Total
Age (years), mean (SD)			64.8 (11.1)		63.2 (10.4)		64.0 (10.8)
**Sex, n (%)**							
	Female	47 (18.8)		38 (14.8)		85 (16.8)	
	Male	203 (81.2)		218 (85.2)		421 (83.2)	
**Ethnicity, n (%)**							
	Australian or New Zealander	132 (52.8)		135 (52.7)		267 (52.8)	
	Asian	51 (20.4)		42 (16.4)		93 (18.4)	
	Other	67 (26.8)		79 (30.9)		146 (28.9)	
**Marital status, n (%)**							
	Married or de facto partnership	185 (74)		183 (71.5)		368 (72.7)	
	Single, separated, or widowed	65 (26)		73 (28.5)		138 (27.3)	
Years of formal education, mean (SD)			13.4 (4.1)		13.8 (4.4)		13.6 (4.2)
**Employment status, n (%)**							
	Working full time	84 (33.6)		88 (34.4)		172 (34)	
	Working part-time	18 (7.2)		19 (7.4)		37 (7.3)	
	Not working	148 (59.2)		149 (58.2)		297 (58.7)	
**History of CHD, n (%)**							
	All CHD	250 (100)		256 (100)		506 (100)	
	Myocardial infarction	149 (59.6)		160 (62.5)		309 (61.1)	
	CABGs^a^	74 (29.6)		68 (26.6)		142 (28.1)	
	PCI^b^	154 (61.6)		163 (63.7)		317 (62.6)	
History of type 1 diabetes, n (%)			3 (1.2)		1 (0.4)		4 (0.8)
History of type 2 diabetes, n (%)			125 (50)		121 (47.3)		246 (48.6)
Family history of CHD, n (%)			151 (60.6)^c^		172 (67.7)^d^		323 (64.2)^e^
Family history of diabetes, n (%)			112 (44.8)		124 (49.2)^f^		236 (47)^g^
**Referral source, n (%)**							
	Specialist or hospital	191 (76.7)^c^		201 (78.5)		392 (77.5)	
	GP^h^ or community	58 (23.3)^c^		55 (21.5)		113 (22.3)	
Current smoker, n (%)			26 (10.5)^i^		24 (9.5)^f^		50 (10)^j^
Current drinker, n (%)			134 (54.3)^i^		158 (62.7)^f^		292 (58.5)^j^
Exercises regularly, n (%)			103 (41.7)^i^		113 (44.8)^f^		216 (43.3)^j^
Total physical activity (MET^k^ minutes per week), mean (SD)			1834 (2748)		2185 (3874)		2010 (3361)
SBP^l^ (mm Hg), mean (SD)			128.8 (17.5)		126.6 (17.6)		127.7 (17.5)
DBP^m^ (mm Hg), mean (SD)			77.0 (11.4)		76.0 (10.3)		76.5 (10.9)
BMI (kg/m^2^), mean (SD)			30.0 (6.2)		31.2 (6.3)		30.6 (6.3)
Waist circumference (cm), mean (SD)			106.0 (14.8)		109.1 (16.2)		107.5 (15.6)
Heart rate, mean (SD)			71.0 (12.2)		69.9 (11.6)		70.4 (11.9)
LDL^n^ cholesterol (mmol/L), mean (SD)			1.9 (0.8)		1.9 (0.9)		1.9 (0.9)
HDL^o^ cholesterol (mmol/L), mean (SD)			1.1 (0.3)		1.1 (0.3)		1.1 (0.3)
Total cholesterol (mmol/L), mean (SD)			3.9 (1.1)		3.9 (1.1)		3.9 (1.1)
Triglycerides (mmol/L), mean (SD)			2.0 (2.3)		1.8 (1.2)		1.9 (1.8)
Fasting glucose (mmol/L), mean (SD)			7.6 (3.2)		7.1 (2.7)		7.3 (3.0)
**Antihypertensive medication, n (%)**							
	ACE^p^ inhibitors	92 (36.8)		98 (38.3)		190 (37.5)	
	Angiotensin 2 receptor blockers	66 (26.4)		74 (28.9)		140 (27.7)	
	Thiazide diuretics	20 (8)		23 (9)		43 (8.5)	
	β-blockers	137 (54.8)		149 (58.2)		286 (56.5)	
	Calcium channel blockers	52 (20.8)		65 (25.4)		117 (23.1)	
	Other antihypertensives	36 (14.4)		44 (17.2)		80 (15.8)	

^a^CABG: coronary artery bypass graft.

^b^PCI: percutaneous coronary intervention.

^c^n=249.

^d^n=254.

^e^n=503.

^f^n=252.

^g^n=502.

^h^GP: general practice.

^i^n=247.

^j^n=499.

^k^MET: metabolic equivalent of task.

^l^SBP: systolic blood pressure.

^m^DBP: diastolic blood pressure.

^n^LDL: low-density lipoprotein.

^o^HDL: high-density lipoprotein.

^p^ACE: angiotensin-converting enzyme.

**Table 6:** Primary and secondary outcomes at the 6-month assessment for participants with coronary heart disease (n=453).

			Control (n=225)^a^		Intervention (n=228)^a^		Adjusted mean difference^b^ or relative risk^c^ (95% CI)		*P* value
**Primary outcome**									
	Systolic blood pressure (mm Hg), adjusted mean (95% CI)	127.7 (125.8 to 129.6)		128.5 (126.6 to 130.4)		0.8 (−1.9 to 3.5)^b^		.55	
**Secondary outcomes**									
	BMI (kg/m^2^), adjusted mean (95% CI)	30.3 (30.1 to 30.5)		30.2 (29.9 to 30.4)		−0.1 (−0.4 to 0.2)^b^		.38	
	Diastolic blood pressure (mm Hg), adjusted mean (95% CI)	76.4 (75.2 to 77.6)		76.6 (75.4 to 77.8)		0.1 (−1.6 to 1.9)^b^		.88	
	Waist circumference (cm), adjusted mean (95% CI)	106.2 (105.3 to 107.2)		106.5 (105.5 to 107.5)		0.3 (−1.1 to 1.7)^b^		.68	
	LDL^d^ cholesterol (mmol/L), adjusted mean (95% CI)	1.7 (1.6 to 2.0)		1.9 (1.9 to 2.0)		0.1 (0.0 to 0.3)^b^		.10	
	Fasting glucose (mmol/L), adjusted mean (95% CI)	6.9 (6.5 to 7.3)		6.9 (6.6 to 7.3)		0.0 (−0.5 to 0.6)^b^		.88	
	Total physical activity (MET^e^ minutes per week), adjusted mean (95% CI)	1744 (1402 to 2089)		2108 (1766 to 2450)		364 (−121 to 849)^b^		.14	
	Fruit consumption (servings per week), adjusted mean (95% CI)	10.8 (9.9 to 11.8)		10.9 (10.0 to 11.9)		0.1 (−1.2 to 1.4)^b^		.90	
	Vegetable consumption (servings per week), adjusted mean (95% CI)	15.7 (14.2 to 17.2)		16.7 (15.2 to 18.3)		1.1 (−1.1 to 3.2)^b^		.34	
	Fish consumption (grams per week), adjusted mean (95% CI)	196.0 (168.9 to 223.3)		237.0 (209.0 to 264.0)		41.0 (2.6 to 79.4)^b^		.04	
	Meals not prepared at home eaten per week, adjusted mean (95% CI)	1.6 (1.4 to 1.9)		1.8 (1.5 to 2.0)		0.1 (−0.2 to 0.5)^b^		.45	
	Using butter or ghee for cooking, n (%)	163 (79.1)^f^		148 (71.2)^g^		0.9 (0.8 to 1.0)^c^		.04	
	Continued current smokers, n (%)	16 (76.2)^h^		12 (63.2)^i^		0.8 (0.6 to 1.3)^c^		.49	
	Quality of life: physical component summary, adjusted mean (95% CI)	46.6 (45.6 to 47.7)		46.9 (45.9 to 48.0)		0.3 (−1.2 to 1.8)^a^		.67	
	Quality of life: mental component summary, adjusted mean (95% CI)	49.2 (48.4 to 50.0)		49.5 (48.7 to 50.3)		0.3 (−0.8 to 1.4)^b^		.58	
	Depression score (PHQ-9^j^), adjusted mean (95% CI)	5.3 (4.8 to 5.9)		5.0 (4.4 to 5.6)		−0.3 (−1.1 to 0.5)^b^		.42	
	Missed medication on at least one of the last 30 days, n (%)	67 (33)^k^		53 (25.5)^g^		0.8 (0.6 to 1.0)^c^		.05	

^a^Number of participants with data available for the primary outcome.

^b^Adjusted mean difference.

^c^Relative risk.

^d^LDL: low-density lipoprotein.

^e^MET: metabolic equivalent of task.

^f^n=206.

^g^n=208.

^h^n=21.

^i^n=19.

^j^PHQ-9: Patient Health Questionnaire–9.

^k^n=203.

### Process Measures

In total, 76.8% (344/448) of the intervention participants responded to the feedback questionnaire. Most respondents reported that the SMS text message support program was useful (298/344, 86.6%); easy to understand (336/344, 97.7%); and appropriate in terms of language, number of messages, and program length (Table 7). There was a high level of engagement, as reflected by the 92.4% (318/344) who read at least three-quarters of their messages and the 54.4% (187/344) who showed the messages to family and friends. Furthermore, most respondents (217/344, 63.1%) agreed that the messages motivated change, and 59.3% (204/344) indicated that the messages reminded them to take their medication (Table 7).

**Table 7:** Summary of utility and perceived acceptability of SMS text message support among 334 intervention participants. Response options were “Strongly agree,” “Agree,” “Neutral,” “Disagree,” and “Strongly disagree” for the first 6 items and final item. We report the proportion for “Agree” and “Strongly agree.” For the first 3 questions regarding message characteristics, we report the percentage of participants who answered.

Process Grouping	Question	Agree or Strongly Agree
Usefulness and understanding	Found messages useful Messages were easy to understand	298/344 (86.6%) 336/344 (97.7%)
Influence on motivation and behavior change	Messages motivated change Diet more healthy because of messages Exercise increased because of messages Messages reminded them to take their medicines	217/344 (63.1%) 209/344 (60.8%) 168/344 (48.8%) 204/344 (59.3%)
Message saving and sharing	Read at least three-fourths of messages Deleted messages Saved messages Showed messages to family or friends Forwarded messages to family or friends	318/344 (92.4%) 47/344 (13.7%) 273/344 (79.3%) 187/344 (54.4%) 41/344 (11.9%)
Appropriate message characteristics	Message language was appropriate Number of messages per week was appropriate Program length was appropriate Time of day messages received was appropriate	311/344 (90.4%) 300/344 (87.2%) 295/344 (85.8%) 258/344 (75.0%)

### Focus Groups

A total of 4.9% (16/325) men and 1.6% (2/129) women from the intervention arm, with a mean age of 62.4 (SD 10.3) years, participated in the focus groups after completing the 6-month program. Thematic analysis demonstrated that participants identified 5 enablers in the program: active reminders to lead a healthy lifestyle, encouragement of dietary changes, encouragement of more physical activity, the ability to share the messages with friends and family, and the feeling of support that the program offered. There was consensus that the content of the messages was appropriate and pitched at the right level. The content on physical activity and diet was considered particularly helpful. Responses illustrating these themes are provided in Table S3 in Multimedia Appendix 1. The 2 main barriers identified were the unidirectional nature of the messages—and, therefore, the lack of interaction with a health professional—and the fact that the messages were insufficiently detailed.

### Adverse Events

There were 6.4% (29/454) of adjudicated hospital events among the intervention participants and 6.3% (28/448) among the control participants (*P*=.93). There were 9 deaths—5 (56%) in the intervention group and 4 (44%) in the control group. No adverse events or deaths were deemed related to the intervention.

## Discussion

### Principal Findings

Maintaining a connection with patients with chronic diseases is a challenge, but it is an important aspect of care as self-management support can prevent or moderate exacerbations. The SupportMe study is the largest RCT to date of an SMS text messaging program providing support for healthy lifestyle and chronic disease self-management and with the largest number of people with diabetes. Novel aspects of this study include the customization of the program to suit people with one or both of the diseases that were the focus—T2D and CHD—and the establishment of the study within the context of a health district–wide chronic disease integrated care program. The study indicates that a program to support patients with chronic diseases using educational and motivational messages is feasible, well received, and engaging. Widespread implementation is perceived to be beneficial, but as illustrated by the lack of an effect on the primary outcome (SBP) as well as secondary outcomes such as HbA_1c_ level, implementation cannot assume a significant or immediate impact on clinical risk factors.

### Findings in Relation to the Literature

Previous studies using SMS text messaging suggest that this may be effective in supporting patient management and improving clinical risk factors. The earlier TEXT ME study delivered a similar SMS text messaging program to participants with CHD (messages nearly identical to those for participants with CHD in SupportMe) and demonstrated improvements in multiple clinical risk factor measures, including LDL-C level, BP, BMI, physical activity, and smoking cessation [9]. A meta-analysis of early small studies showed that SMS text messaging programs for people with T2D achieved an overall reduction in HbA_1c_ level of 0.38% (4 mmol/mol) [13]. Recent studies with large cohorts—Self-Management Support for Blood Glucose (SMS4BG), Cardiovascular Health and Text Messaging-Diabetes Mellitus (CHAT-DM), and Rapid Education/Encouragement and Communications for Health—have demonstrated similar small improvements in HbA_1c_ level of 0.3% to 0.4% (3-4 mmol/mol) [14-16]. However, another contemporary texting program for people with diabetes in Australia also failed to demonstrate an improvement in HbA_1c_ level, although there was a similarly high level of program satisfaction and acceptability [27].

The lack of improvement in clinical metrics in SupportMe may be due to baseline metrics that were within or close to the guideline targets among many participants. The mean baseline SBP was 128.9 (SD 17.2) mm Hg, and the mean LDL-C level was 2.0 (SD 0.9) mmol/L. In Australian guidelines, for most people with hypertension, a BP target of <140/90 mm Hg is recommended [28]. For people at high cardiovascular risk, an LDL-C level of <2.0 mmol/L is recommended [21]. Therefore, many participants in SupportMe were already at the target BP and lipid levels at baseline. In TEXT ME, after 6 months of intervention, the SBP was 128 mm Hg, and the LDL-C level was 2.0 mmol/L, which are similar to our baseline measures [9]. Only 8.2% (74/902) of the participants in our study were current smokers compared with 53% in TEXT ME [9] and 41% and 15% in the CHAT-DM and SMS4BG studies, respectively [14,16]. The baseline HbA_1c_ level in SupportMe was 8.4% (68 mmol/mol) compared with 9.8% (84 mmol/mol) in SMS4BG [14]. Participants in SupportMe undertook 1898 MET minutes per week of physical activity compared with 380 MET minutes per week in TEXT ME and 1386 MET minutes per week in CHAT-DM [9,16]. These factors suggest that the SupportMe cohort was more focused on healthy lifestyle behaviors or received better disease management than participants in previous studies. Therefore, there was much less room for the SMS text messages to achieve further improvement.

The involvement of specialist and hospital teams as part of the WSICP may have contributed to the favorable baseline metrics in SupportMe. GPs who referred community patients had greater engagement in the management of T2D and CHD and were supported by care facilitators who helped with guideline management of patients with these chronic diseases [18]. Thus, participants were already receiving high-level professional care and support. This is reflected in the decrease in HbA_1c_ level of 0.6% (7 mmol/mol) in the control arm of SupportMe, which is similar to that of the intervention arm of the SMS4BG study and greater than that of the intervention arm in the CHAT-DM study [14,16].

The age of the SupportMe participants, particularly with respect to the participants with diabetes (mean 60.2, SD 11.4 years), might be another factor that influenced the results. The mean age of participants in SMS4BG, Rapid Education/Encouragement and Communications for Health, and CHAT-DM was 47, 56, and 60 years, respectively, so our participants were among the oldest in these studies. The SupportMe CHD participants (mean age 64.0, SD 10.8 years) were also older than the TEXT ME participants (mean age 57 years). Perhaps older patients are less responsive to this mode of engagement as they are less familiar with the regular use of this technology, although our evaluation did indicate that most of the messages were read. It would be interesting to test a similar SMS text messaging program for a younger population with multiple chronic diseases.

Some programs have incorporated return messages from participants, which may improve interaction and engagement [14,15]. Our focus groups identified the lack of bidirectional messaging as a barrier. A meta-regression analysis of telemedicine trials for diabetes found that interventions that included provider-to-patient messaging and those in which providers adjusted medication in response to data from patients were more effective [29]. However, this requires considerably more resources and day-to-day clinician involvement. Widespread implementation of such a model is not feasible in most health systems. The focus groups also revealed a desire for more detailed information, which was difficult to provide in a short SMS text message. Future programs with internet-connected phones can overcome this with increased use of embedded web links.

We found better self-reported medication adherence with the SMS text messaging intervention. This is consistent with other studies, with a meta-analysis of 16 RCTs showing that SMS text messaging is effective in increasing medication adherence independently of whether 2-way communication is used [8]. Perhaps the relatively large number of messages related to medication in SupportMe (16 to 18 depending on health condition, with an additional 5 for those treated with insulin) contributed to an improvement in medication adherence. Notably, the reduction in missed medication was significant in the CHD subgroup but not in the diabetes subgroup. It is possible that complex diabetes medication regimens are a greater barrier to full adherence. Improving medication adherence is nonetheless challenging, with a similar trial conducted by our group failing to demonstrate that supportive SMS text messages improve medication adherence among patients following acute coronary syndromes [30].

Although we did not find an improvement in short-term clinical metrics, the high levels of engagement and acceptability may have other positive implications. Patient engagement and activation have been linked to better health outcomes and patient experience and reduced health care costs [31]. Engaged patients are more likely to participate in healthy behaviors and seek and use health information [31]. For the population involved in SupportMe with T2D or CHD, this may translate into less long-term demand on the highly specialized and limited workforce in these fields.

A limitation of this study was the failure to reach the planned sample size for the primary outcome. However, it is unlikely that recruitment of an extra 98 participants would have changed the results to be significantly in favor of the intervention. It was also disappointing that primary outcome data could not be obtained for 10.5% (95/902) of the participants. Although many participants were very engaged, there was also a group in which engagement and follow-up were challenging. Arguably, these are the patients who most require support, and SMS text messaging may not be the optimal means of achieving this. Finally, we accepted blood test results ordered from a range of clinicians performed in different laboratories. In some cases, clinical metrics for participants who were unable to return for review were obtained from their primary physicians. Thus, laboratory testing and some physical measurements were not standardized.

The question of whether the SMS text messages could have been better formulated is a vital one. The messages were developed through a proven and rigorous process [20,32], and many had been used in a previous trial where SMS text messaging was successful in improving clinical metrics [9]. We have also recently demonstrated that similar healthy lifestyle messages prompt an increase in physical activity among women at risk of diabetes [33]. Nonetheless, further development and testing of messages for any similar program would be desirable.

### Conclusions

Mobile phone ownership is very high in many countries, even in low- and middle-income countries. This enables the delivery of health messages to a large population group, overcoming access barriers at a low cost. The telecommunications cost of delivering 4 SMS text messages per week for 6 months was in the order of Aus $6 (US $4.05) per participant. Thus, there is increasing interest in digital health interventions. SMS text messaging has also been trialed as an adjunct to routine care for people with a range of other chronic diseases other than CHD and diabetes, including chronic kidney disease [34], asthma [35], hypertension [36], and depression [37]. However, these studies focused on single risk factors or conditions, yet chronic diseases are highly clustered. A novel aspect of our program is that we were able to customize it for people with 1 or 2 chronic diseases. As the population with multiple comorbidities increases, having a single SMS text messaging program that has modules for several or a mix of different chronic conditions is preferable to the patient needing to enroll in multiple SMS text messaging programs for different conditions. SupportMe has demonstrated the feasibility of this. It would be important to further test SMS text messaging programs for multiple chronic diseases in populations that are less well supported and have less access to professional care.

A key learning from this study is that SMS text messaging programs vary in their effectiveness and utility depending on the precise population and context. Our results do not indicate that SMS text messaging programs are generally ineffective in improving clinical metrics among people with T2D or CHD but that the goal may need to be tailored to the situation. Within an integrated care environment where there is already high-level care, SMS text messaging may provide other less tangible benefits. The co-design process of the SupportMe intervention with a multidisciplinary team and consumers facilitated the development of processes and content for the program as a whole. The high acceptability of the program across participants with multiple conditions indicates that the program can complement routine care by improving the patient experience and sense of engagement, which are important aspects of integrated care.

## Appendix

Multimedia Appendix 1 Number of SMS text messages available for each health condition by topic addressed, questions used as a guide to facilitate focus group discussions, and example quotations illustrating the 5 major themes identified as enablers in SupportMe focus groups, respectively.

Multimedia Appendix 2 SupportMe diet questionnaire.

Multimedia Appendix 3 eCONSORT checklist.

## Abbreviations

BP: blood pressure
CHAT-DM: Cardiovascular Health and Text Messaging-Diabetes Mellitus
CHD: coronary heart disease
DBP: diastolic blood pressure
GP: general practitioner
HbA_1c_: glycated hemoglobin
LDL-C: low-density lipoprotein cholesterol
MET: metabolic equivalent of task
RCT: randomized controlled trial
REDCap: Research Electronic Data Capture
SBP: systolic blood pressure
SMS4BG: Self-Management Support for Blood Glucose
T2D: type 2 diabetes
TEXT ME: Tobacco, Exercise, and Diet Messages
WSICP: Western Sydney Integrated Care Program
WSLHD: Western Sydney Local Health District
