# Supplementary material for: Effect of Mobile Phone Text Messaging Self-Management Support for Patients With Diabetes or Coronary Heart Disease in a Chronic Disease Management Program (SupportMe) on Blood Pressure: Pragmatic Randomized Controlled Trial
Source: J Med Internet Res. 2023 Jun 16;25:e38275. doi: 10.2196/38275 (PMC10337246; doi:10.2196/38275)
Supplement: Multimedia Appendix 1 [file jmir_v25i1e38275_app1.docx]

**Multimedia Appendix 1: Supplementary Tables**

This is a Multimedia Appendix to a full manuscript published in the J Med Internet Res. For full copyright and citation information see http://dx.doi.org/10.2196/jmir.xxxx

Table S1. Number of text messages available for each health condition, by topic addressed.

|  | Health Condition | | |
| --- | --- | --- | --- |
|  | Diabetes | Coronary Heart Disease | Diabetes and Coronary Heart Disease |
| Cardiovascular risk factors | 10 | 13 | 11 |
| Healthy weight and eating | 8 | 12 | 15 |
| Medication | 16 | 18 | 16 |
| Health and support services | 8 | 11 | 9 |
| Psychosocial | 8 | 4 | 5 |
| Fruit and vegetables | 16 | 30 | 19 |
| Carbohydrate | 7 | 1 | 6 |
| Salt | 7 | 14 | 10 |
| Fats, meat, protein | 12 | 13 | 13 |
| Drinks | 5 | 4 | 5 |
| Physical activity facts and guidelines | 8 | 6 | 8 |
| Physical activity tips and reminders | 33 | 33 | 33 |
| Smoking | 37 | 38 | 38 |
| Diabetes monitoring | 15 | 0 | 11 |
| Hypoglycaemia | 3 | 0 | 1 |
| Diabetes complications | 10 | 0 | 8 |
| Insulin | 5 | 0 | 5 |
| Total messages available | 208 | 197 | 213 |

Table S2. Questions used as a guide to facilitate focus group discussions

| Domain | Probes |
| --- | --- |
| General | 1. What did you think of the SUPPORTME text message program?  2. Do you think it is helpful to have information delivered via mobile phone text messages?  3. Do you receive other information via mobile phone text message?  4. Do you use your mobile phone in other ways to receive information about health?  5. Did you find the messages allowed you to improve your diabetes control? |
| Message delivery | 6. What did you think of the way the messages were delivered?  7. How many messages did you receive per week?  8. What did you think of the frequency of the messages?  9. What did you think of the time that the message was delivered? |
| Message content | 10. Did you find the content of the text messages were helpful? Did you find the messages motivated you?  11. Did you find the messages a helpful source of information?  12. Did you find the messages annoying?  13. What messages did you like the most?  14. Did you show anyone the messages? |
| Messages and behavioural change | 15. Did you think the messages change your behaviours in any way? E.g. lifestyle, treatments, access to medical services?  16. Did the messages prompt you to look at other sources of information or ask other questions of your health practitioners?  17. What do you think it was about the messages that made you change your behaviours?  18. Do you think you were better equipped to manage your diabetes as a result of the messages? |
| Program improvement | 19. How would you improve the program?  20. If you were to roll this program out across Australia, what changes or improvements might you make?  21. Do you think it could be used for other health programs?  22. Do you think it should be prescribed by health practitioners or hospitals or self-prescribed through a web page? |
| General comments: | 23. Do you have any other comments about the program? |

Table S3. Example quotations illustrating the 5 major themes identified as enablers in SupportMe focus groups

| Theme | Participant | Quote |
| --- | --- | --- |
| Reminder | 68 year old male, CHD | *‘I think it’s a nice friendly reminder, it’s not telling you what to do, it’s just making suggestions.’* |
|  | 58 year old female, T2D | *‘I found it very helpful. Because it reminded me and kept me motivated. A message would come through and say eat this or do that, and you think, yeah, you have to take time out for yourself. But you’ve got so much going on in your life, that it’s not that you’ve forgotten but it just jogs your memory. Eat healthy, do this, look after yourself. I liked it and I always looked forward to it. I’ve got no complaints about it.’* |
| Diet | 41 year old male, CHD | *‘The one thing I’ve changed is in the mornings I used to have eggs, toast and bacon – that’s gone. I still do that but maybe once a month… it is a treat.’* |
|  | 58 year old male, CHD/T2D | *‘Don’t eat biscuits - we all know that. I think twice now, it’s changed me.’* |
| Physical activity | 62 year old male, CHD/T2D | *‘Well it inspires you… I better go do some walking.’* |
|  | 67 year old male, CAD | *'...it’s a good reminder. Occasionally there are some things about exercise, reminded me to get out and go and do it'* |
| Lifestyle change | 61 year old male, CHD/T2D | *‘Overall it helped me to keep focused on controlling my dietary and general health like walking, food portion control and the dangers of what diabetes does to you if left unchecked.’* |
|  | 41 year old male, CHD | *With the messages that come up every so often, it keeps your mind over there. You are always thinking about it, and it’s not just about what it’s saying on the message. The fact that the message came through is just reminding you to think healthy. So, not necessarily about that content, but about making good choices. When it comes to food, we walk past [McDonald’s] and everyone is like yeah I need to go and get a burger. But because you are getting these messages, you are thinking, that’s not what Support Me would suggest’* |
| Message sharing | 58 year old male, CHD/T2D | *‘I have them all saved… and the kids read it and they have all lost weight. We don’t have biscuits now, we have fresh fruit’* |

CHD = coronary heart disease, T2D = type 2 diabetes
