# Supplementary figures and images for: Effect of Mobile Phone Text Messaging Self-Management Support for Patients With Diabetes or Coronary Heart Disease in a Chronic Disease Management Program (SupportMe) on Blood Pressure: Pragmatic Randomized Controlled Trial
Source: J Med Internet Res. 2023 Jun 16;25:e38275. doi: 10.2196/38275 (PMC10337246; doi:10.2196/38275)

## SupportMe Diet Questionnaire


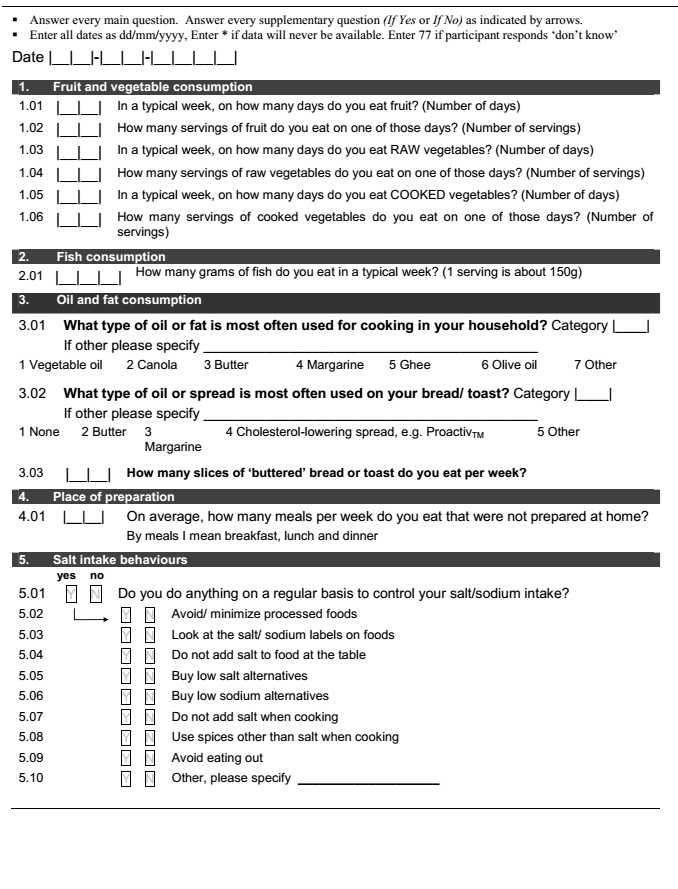

Supplement: Multimedia Appendix 2 [file jmir_v25i1e38275_app2.docx]
